# Development of a National Clinical Registry for CABG Surgery in Saudi Arabia—The SCAR Project

**DOI:** 10.3390/jcm15135114

**Published:** 2026-07-01

**Authors:** Mohsin Murshid, Osama Abdulrahman, Uthman AlUthman

**Affiliations:** 1 Division of Cardiothoracic Surgery, King Faisal Specialist Hospital and Research Center, Jeddah 23433, Saudi Arabia; mohsin.murshid90@outlook.com; 2 Division of Cardiac Surgery, King Abdullah Medical City, Makkah 24246, Saudi Arabia; osama1999su@gmail.com

**Keywords:** coronary artery bypass grafting, clinical quality registry, cardiac surgery, outcomes monitoring, quality improvement, Saudi Arabia

## Abstract

**Background:** Coronary artery disease is a leading cause of morbidity and mortality in Saudi Arabia. No national mechanism exists for standardized CABG outcome surveillance. The Saudi CABG Audit and Registry (SCAR) was established to address this gap. **Methods:** SCAR is a prospective multicenter clinical quality registry enrolling consecutive patients undergoing isolated or combined CABG across Saudi Arabia. The dataset captures more than 100 variables spanning demographics, comorbidities, operative details, in-hospital outcomes, and longitudinal follow-up to one year. Primary outcomes include in-hospital mortality, 30-day major adverse cardiac and cerebrovascular events (MACCE), deep sternal wound infection, and unplanned reoperation. **Results:** The registry incorporates structured follow-up at 30 days, 3 months, 6 months, and one year, governed through three national committees overseeing data quality and dissemination. A four-tier analytical framework progresses from descriptive analyses through risk-adjusted benchmarking, validation of international risk models, and future development of a locally derived risk prediction model. Implementation commences with a pilot phase enrolling 100–150 consecutive procedures across four centers over three months. **Conclusions:** SCAR represents the first dedicated national CABG registry in Saudi Arabia. Through risk-adjusted benchmarking and structured feedback, the registry may support quality improvement, multicenter research, healthcare planning, and development of locally relevant risk stratification tools.

## 1. Background

Cardiovascular disease remains the leading cause of mortality worldwide, accounting for an estimated 17.9 million deaths annually [1]. In Saudi Arabia, the burden continues to rise because of the high prevalence of obesity, diabetes mellitus, hypertension, dyslipidemia, and sedentary lifestyles [2,3]. Coronary artery disease remains the most common manifestation of cardiovascular disease and is a major contributor to morbidity, mortality, and healthcare expenditure within the Kingdom. Coronary artery bypass grafting (CABG) remains a cornerstone revascularization strategy for patients with complex multivessel disease, diabetes mellitus, and impaired ventricular function. Despite advances in medical therapy and percutaneous coronary intervention, CABG continues to provide substantial survival and symptomatic benefits in appropriately selected patients with advanced coronary artery disease.

Advances in cardiac surgery, anesthesia, and perioperative care have substantially improved CABG outcomes over recent decades. However, accurate evaluation of surgical performance and patient outcomes requires comprehensive, standardized, longitudinal data collection systems capable of capturing perioperative variables, postoperative complications, and long-term outcomes [4]. To address this need, several countries have established national cardiac surgery registries, including the Society of Thoracic Surgeons (STS) Adult Cardiac Surgery Database in the United States, the European E-CABG Registry, and the National Adult Cardiac Surgery Audit (NACSA) in the United Kingdom [5,6,7]. These registries have supported risk-adjusted benchmarking, quality improvement initiatives, public reporting, and development of evidence-based clinical guidelines. For example, participation in the STS database has been associated with reductions in operative mortality following isolated CABG from approximately 2.5% to approximately 1.9% in recent years [5]. These registries have demonstrated the value of structured clinical data collection in identifying practice variation, supporting quality assurance, and informing healthcare policy.

## 2. Rationale

Despite the high burden of coronary artery disease and the widespread performance of CABG procedures across tertiary cardiac centers in Saudi Arabia, there is currently no national cardiac surgery registry capable of systematically capturing perioperative and long-term outcomes. Existing data are largely limited to isolated single-center experiences, restricting opportunities for national benchmarking, validation of international risk prediction models, development of locally relevant risk stratification tools, and data-driven healthcare planning. Although initiatives such as the SPACE registry have demonstrated the feasibility of large-scale cardiovascular data collection within Saudi Arabia [8], no equivalent national framework currently exists for surgical coronary revascularization.

The Saudi CABG Audit and Registry (SCAR) was therefore developed as a prospective multicenter clinical quality registry for patients undergoing CABG surgery across Saudi Arabia. The registry incorporates internationally validated parameters and definitions, including EuroSCORE II [9], SYNTAX score [10], Kidney Disease: Improving Global Outcomes (KDIGO) criteria [11], and standardized complication definitions, while also integrating locally relevant domains such as frailty, cardiac rehabilitation, healthcare access, and patient-reported outcomes. The dataset incorporates key echocardiographic and cardiovascular imaging parameters used in contemporary perioperative risk assessment, reflecting the established and growing role of multimodality imaging in cardiovascular risk stratification and outcome prediction [12]. Validated quality-of-life instruments, including the EuroQol Five-Dimension Five-Level questionnaire (EQ-5D) and the Short Form 36 Health Survey (SF-36), together with return-to-work and functional recovery measures, are incorporated to support patient-centered outcome assessment. In addition, referral to and participation in cardiac rehabilitation programs will be captured because of their established association with improved functional recovery and long-term cardiovascular outcomes.

Through standardized national data collection, structured audit mechanisms, risk-adjusted benchmarking, and institutional feedback, SCAR may support quality improvement initiatives, multicenter cardiovascular research, and evidence-informed healthcare planning. The registry is also intended to provide a foundation for future expansion into other cardiac surgical procedures and facilitate Saudi Arabia’s participation in international cardiovascular surgical collaborations. This study is registered in ClinicalTrials.gov with the identifier NCT07316777.

## 3. Study Design

SCAR is a prospective, observational, multicenter clinical quality registry designed to capture detailed perioperative and long-term outcome data for patients undergoing CABG in Saudi Arabia. The registry is intended to support risk-adjusted benchmarking, quality improvement initiatives, and the generation of real-world evidence to inform clinical practice, national cardiac surgery policy, and multicenter research. Its primary objectives are to systematically document patient characteristics, operative strategies, and short- and long-term outcomes while facilitating national performance monitoring and audit activities.

## 4. Current Implementation Status

At the time of manuscript submission, SCAR remains in the preparatory implementation phase. Four tertiary cardiac surgery centers are currently under review for pilot participation, with institutional collaboration agreements progressing through local institutional review board (IRB) review and site onboarding processes at each prospective center. The names of participating institutions will be confirmed and disclosed following completion of local IRB review and formal execution of collaboration agreements. Patient recruitment and data collection have not yet commenced.

## 5. Pilot Phase

Registry implementation will commence through an initial pilot phase involving four selected tertiary cardiac surgery centers. The pilot phase is expected to enroll approximately 100–150 consecutive CABG procedures over a period of three months and is intended to evaluate operational feasibility, data completeness, follow-up capture rates, data quality processes, user experience, and platform performance before broader national implementation.

Key pilot success indicators will include a data completeness rate of at least 90% across mandatory fields, a 30-day follow-up capture rate of at least 80%, adherence to standardized variable definitions, usability of the electronic platform, and overall operational feasibility. Predefined thresholds for these indicators must be met before progression to national rollout is initiated. National expansion is anticipated to begin within 12 to 18 months of pilot phase completion, with the registry aiming to capture a substantial and progressively increasing proportion of CABG procedures performed at tertiary cardiac surgery centers across Saudi Arabia. Findings from the pilot phase will be used to refine registry procedures and inform the timeline and scope of subsequent national expansion.

## 6. Study Population

Patients undergoing coronary artery bypass grafting (CABG) will be enrolled consecutively from participating tertiary cardiac surgery centers across Saudi Arabia.

### 6.1. Inclusion Criteria

1. Age ≥ 18 years.
2. Patients undergoing isolated CABG.
3. Patients undergoing CABG combined with concomitant cardiac procedures (e.g., valve repair or replacement).
4. Procedures performed using on-pump or off-pump techniques.
5. Elective, urgent, or emergent procedures.
6. Open, minimally invasive, or robotic-assisted surgical approaches.

### 6.2. Exclusion Criteria

1. Patients aged < 18 years.
2. Redo CABG procedures in which the primary operative record is unavailable or irretrievable.

For the purposes of this registry, a primary operative record will be considered unavailable or irretrievable if the original operative documentation and essential perioperative data required for baseline characterization and risk adjustment cannot be obtained from institutional records despite reasonable retrieval attempts.

Participation in the registry will not influence clinical decision-making, perioperative management, or therapeutic strategies, which will remain entirely at the discretion of the treating clinical team according to institutional practices and current clinical guidelines.

All eligible CABG procedures will be entered consecutively to maximize representativeness and minimize selection bias. Participating centers will be encouraged to maintain screening logs documenting all eligible procedures and reasons for non-enrollment where applicable, thereby supporting assessment of case capture rates and registry representativeness. Where local consent requirements result in patient non-participation, this will be recorded in the screening log to allow evaluation of any systematic differences between enrolled and non-enrolled cases. Isolated CABG and combined CABG procedures will be analyzed separately where appropriate, with procedure type incorporated into risk-adjustment models and benchmarking analyses.

Informed consent procedures will follow the policies and requirements of participating institutions and reviewing ethics committees. Participating centers will utilize their own locally approved consent forms and consent procedures where required by institutional policies or ethics committees. Where written informed consent is required, patients will be provided with information regarding the purpose of the registry, the nature of the collected data, confidentiality safeguards, and their rights regarding participation and withdrawal. Variability in local consent requirements across participating institutions may influence patient enrollment procedures and could potentially affect case capture rates and registry representativeness; this is acknowledged as a limitation of the multicenter design. Registry participation will not affect clinical care or treatment decisions.

## 7. Registry Design

The Saudi CABG Audit and Registry (SCAR) is a national clinical quality registry designed to systematically capture perioperative and long-term outcomes of patients undergoing coronary artery bypass grafting (CABG) across Saudi Arabia. The registry was developed by adapting key structural and operational principles from leading international cardiac surgery registries while incorporating locally relevant clinical and healthcare system considerations. SCAR aims to establish a standardized national platform for outcome monitoring, benchmarking, quality improvement, and multicenter cardiovascular research.

The registry is being developed through a multidisciplinary collaborative framework involving cardiac surgeons, senior residents, data scientists, epidemiologists, health informatics specialists, and health services researchers. This working group has overseen registry design, parameter selection, variable standardization, platform development, and quality assurance processes. SCAR will operate through a fully digital infrastructure with centralized governance to ensure consistency, data integrity, and standardized reporting across participating centers.

Registry variables, definitions, and data collection procedures will undergo periodic review to ensure continued relevance and alignment with evolving evidence, clinical practice, and stakeholder requirements.

## 8. Governance Structure

**Figure 1:**
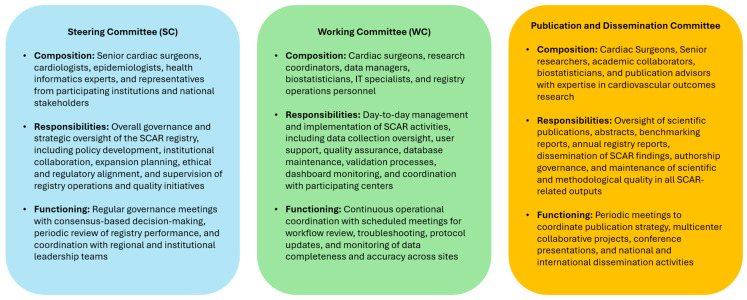
The three committees responsible for governance of SCAR.

The registry will operate under a structured national governance framework designed to ensure scientific integrity, operational efficiency, data quality, and collaborative participation across participating institutions. Oversight of the registry will be conducted through three principal committees: the Steering Committee (SC), the Working Committee (WC), and the Publication and Dissemination Committee (P&DC), as illustrated in Figure 1.

Members of the committees will be nominated by their respective institutions and appointed for renewable one-year terms. Collectively, the committees will oversee protocol adherence, registry operations, institutional coordination, data quality assurance, dissemination activities, implementation of registry policies, and strategic development of the registry.

## 9. Data Access and Use

All participating institutions will enter into formal data-sharing and collaboration agreements outlining governance principles, data confidentiality requirements, publication policies, and procedures for data access and use. Proposals for secondary analyses or sub-studies utilizing SCAR data will undergo committee review and approval based on scientific merit, methodological rigor, feasibility, and alignment with registry objectives.

## 10. Quality Assurance and Oversight

Data quality will be supported through standardized variable definitions, mandatory data fields, electronic validation rules, user training programs, periodic data quality assessments, and source-data verification audits. Participating centers will receive regular feedback regarding data completeness, consistency, follow-up performance, and outcome reporting. Centers demonstrating persistent data quality concerns may undergo targeted retraining, corrective action procedures, and follow-up quality reviews.

Source-data verification audits will be conducted on a random sample of 5–10% of submitted cases at each participating center per audit cycle to assess data completeness, accuracy, and consistency between registry entries and source clinical documentation. A missing data rate exceeding 10% across mandatory fields, or a pattern of systematic inconsistency identified during an audit, will trigger a formal corrective action process comprising targeted retraining, data reconciliation, and a follow-up quality review within 60 days. Data quality indicators, including completeness rates, follow-up capture rates, missing-data frequencies, and validation-error rates, will be monitored periodically by the registry governance structure.

As SCAR is an observational clinical quality registry without interventional components, the Steering Committee will assume responsibility for registry governance, data integrity, and quality assurance oversight during the pilot phase, fulfilling the functions typically discharged by a dedicated Data and Safety Monitoring Board. As the registry transitions to national rollout and the Steering Committee expands to include representatives from additional participating institutions, a formal independent oversight body will be appointed at the appropriate stage of registry maturation. Any significant concerns relating to data quality, governance, or registry operations will be reviewed through the registry’s governance framework and managed according to established quality assurance procedures.

## 11. Data Collection and Follow-Up

Data for SCAR will be collected at standardized intervals beginning at hospital admission and continuing through one year postoperatively. The dataset is organized into domains aligned with international cardiac surgery registry standards while incorporating variables relevant to the Saudi healthcare context. Standardized definitions will be used across participating centers to ensure data consistency, facilitate benchmarking, and enable meaningful national and international comparisons. The complete list of domains, time points, and key variables is presented in Table 1.

Follow-up assessments will be performed at 30 days, 3 months, 6 months, and one year after surgery. Outcome ascertainment will be achieved through a hierarchical approach: outpatient clinic visits and hospital electronic medical records will serve as the primary sources of follow-up data, supplemented by review of readmission records and institutional follow-up databases where available. Direct telephone contact will be used when clinic-based or record-based ascertainment is not achievable. A minimum of three documented contact attempts will be made before a patient is recorded as lost to follow-up. Where available, linkage with national mortality records or Ministry of Health administrative databases may be explored to support outcome ascertainment, particularly for mortality endpoints. Participating centers will be encouraged to maintain accurate patient contact information and verify contact details at each follow-up encounter to minimize loss to follow-up.

Patient-reported outcomes will be assessed using validated Arabic and English versions of the EQ-5D and SF-36 instruments. Baseline assessments will be obtained preoperatively and repeated at 3 months, 6 months, and one year postoperatively to facilitate longitudinal evaluation of functional recovery and health-related quality of life. Administration may be performed in a clinic, by telephone, or electronically according to center capability and patient preference, with efforts made to minimize missing responses through standardized administration protocols. Follow-up data will include survival, major adverse cardiac and cerebrovascular events (MACCE), functional recovery, cardiac rehabilitation referral and participation, return-to-work status, and patient-reported outcome measures.

Cardiac rehabilitation data will include referral status, participation, and program completion where available. Return-to-work status will be assessed among eligible patients and interpreted in the context of age, employment status, and retirement status where applicable.

Both isolated CABG and CABG combined with concomitant cardiac procedures will be included within the registry. Procedure type will be recorded prospectively and incorporated into risk-adjustment models, benchmarking analyses, and outcome reporting to account for differences in operative complexity and baseline risk profiles.

## 12. Objectives and Outcomes

SCAR is designed to establish a national platform for evaluating perioperative and long-term outcomes following coronary artery bypass grafting (CABG) in Saudi Arabia. The registry is based on the premise that standardized nationwide collection of detailed perioperative data will enable identification of patient-, procedure-, and institution-related factors associated with adverse outcomes following CABG surgery.

This study aims to evaluate clinical outcomes, perioperative risk factors, long-term recovery patterns, patient-reported outcomes, and institutional variability among patients undergoing CABG surgery across participating centers in Saudi Arabia.

### 12.1. Primary Objective

To determine the association between preoperative risk factors, intraoperative variables, and postoperative clinical outcomes following CABG surgery, including mortality, major adverse cardiac and cerebrovascular events (MACCE), deep sternal wound infection, and unplanned reoperation.

### 12.2. Secondary Objectives

1. To evaluate perioperative outcomes, including intensive care unit (ICU) stay, duration of mechanical ventilation, hospital length of stay, blood product utilization, and postoperative complications.
2. To determine predictors of postoperative morbidity and mortality in patients undergoing isolated or combined CABG procedures.
3. To assess institutional variability in CABG outcomes using standardized outcome measures and risk-adjusted benchmarking methodologies.
4. To evaluate long-term outcomes, including one-year survival, MACCE, readmissions, return-to-work status, and recovery of baseline functional status following surgery.
5. To assess participation in cardiac rehabilitation programs and their association with postoperative recovery and long-term outcomes.
6. To assess patient-reported quality-of-life outcomes using validated assessment tools.
7. To validate the performance of established international risk prediction models, including EuroSCORE II and the Society of Thoracic Surgeons (STS) risk models, within the Saudi cardiac surgical population.
8. To facilitate multicenter clinical research and quality improvement initiatives through standardized national data collection.

### 12.3. Primary Outcomes

Primary outcomes will include in-hospital mortality, 30-day major adverse cardiac and cerebrovascular events (MACCE), deep sternal wound infection according to Centers for Disease Control and Prevention (CDC) criteria [19], and unplanned reoperation for bleeding, cardiac tamponade, or graft-related technical failure. Thirty-day MACCE will be defined as a composite endpoint of all-cause mortality, stroke, and myocardial infarction. Perioperative myocardial infarction will be defined according to the Type V universal definition [18]. Stroke will be defined as a new focal neurological deficit of presumed vascular origin lasting more than 24 h and confirmed by neuroimaging where available. Unplanned reoperation will be defined as any return to the operating theatre during the index hospitalization for a complication directly related to the index CABG procedure. Acute kidney injury will be defined and staged according to KDIGO criteria [11].

### 12.4. Secondary Outcomes

Secondary outcomes will include ICU and hospital length of stay, duration of postoperative mechanical ventilation, ICU readmission during index hospitalization, all-cause readmission within 30 days, acute kidney injury, and initiation of renal replacement therapy according to KDIGO criteria [11], postoperative arrhythmias requiring intervention, perioperative blood product utilization, and participation in cardiac rehabilitation programs.

Long-term outcomes will include one-year all-cause survival, one-year MACCE, repeat revascularization, return-to-work status or resumption of baseline functional activity, participation in cardiac rehabilitation programs, and longitudinal changes in health-related quality of life. Health-related quality-of-life measures will be assessed using baseline and follow-up EQ-5D and SF-36 patient-reported outcome instruments to facilitate evaluation of recovery trajectories and clinically meaningful changes over time.

A comprehensive data dictionary defining all primary and secondary outcome variables, including standardized definitions, data sources, and adjudication criteria, will be provided to site leads and data entry personnel at each participating center for reference during data collection and registry operations.

## 13. Development of a Saudi CABG Risk Prediction Model

One of the long-term objectives of SCAR is to facilitate validation of existing international risk prediction models and support the future development of a locally derived CABG risk prediction model for the Saudi population. Initially, registry data will be used to evaluate the performance of established risk stratification systems, including EuroSCORE II and the Society of Thoracic Surgeons (STS) risk models, within the Saudi cardiac surgical population. This validation phase will be undertaken as a priority objective once sufficient registry data have been accrued and will precede any consideration of local model development.

Model performance will be assessed using established measures of discrimination and calibration. Discriminatory ability will be evaluated using the area under the receiver operating characteristic curve (AUC) or c-statistic, while calibration will be assessed through calibration plots, observed-to-expected outcome comparisons, and the Hosmer–Lemeshow test or equivalent calibration metrics where appropriate.

Development of a SCAR-derived risk prediction model will be considered only after accrual of a sufficiently large national dataset with adequate outcome event rates to support robust model development and validation. As a minimum threshold, model development will not be initiated until at least 500 outcome events for the primary endpoint of interest have been accrued across participating centers, in accordance with established recommendations for clinical prediction model development. Candidate predictors will include demographic characteristics, comorbid conditions, frailty measures, cardiac disease severity, operative variables, imaging findings, and perioperative laboratory parameters routinely collected within the registry. Variable selection will be guided by clinical relevance, data completeness, and established predictor selection methodologies to avoid overfitting.

Internal validation will be performed using bootstrap resampling with a minimum of 1000 iterations to provide optimism-corrected estimates of model discrimination and calibration. Subsequent temporal validation using later registry data and external validation across participating centers will be undertaken before any consideration of clinical implementation. Model recalibration and updating strategies will be explored as additional data become available over time.

Any resulting model will be intended to complement existing international risk stratification systems rather than replace them. By incorporating local demographic, clinical, and healthcare system characteristics, such a model may improve risk estimation and benchmarking within the Saudi cardiac surgical population.

## 14. Electronic Data Management, Security, and Quality Assurance

**Figure 2:**
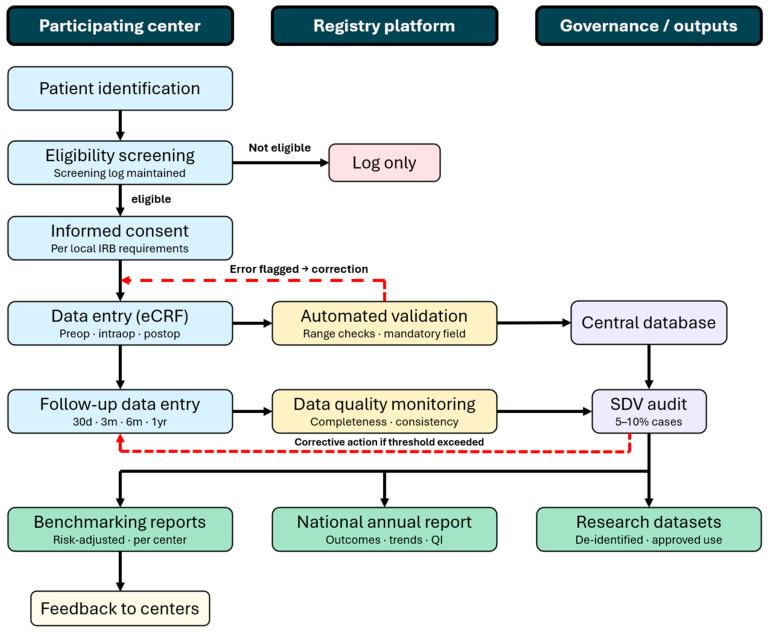
SCAR data flow and quality assurance pathway. Eligible patients are enrolled consecutively, with data entered into the electronic case report form (eCRF) at predefined time points and subjected to automated validation prior to acceptance into the central database. Source-data verification audits are conducted on a random sample of 5–10% of submitted cases per audit cycle. Registry outputs include institutional benchmarking reports, an annual national report, and de-identified research datasets. eCRF, electronic case report form; SDV, source-data verification; QI, quality improvement.

All registry data will be collected and stored using a secure web-based electronic case report form (eCRF) within the SCAR data platform. The registry infrastructure is designed to support standardized multicenter data collection, longitudinal follow-up, audit functionality, real-time analytics, and secure institutional access across participating centers. The platform incorporates structured electronic forms, predefined variable definitions, automated validation rules, integrated dashboards, and standardized reporting tools to facilitate consistent and reliable data capture. The complete data flow pathway, from patient identification through data entry, quality assurance, and output generation, is illustrated in Figure 2.

Personnel involved in registry data collection and submission will undergo standardized training prior to receiving platform access. Training will cover registry objectives, variable definitions, data collection procedures, outcome ascertainment, and data quality requirements. All users will be provided with a comprehensive data dictionary containing standardized definitions, variable descriptions, and data entry instructions. The data dictionary will also be integrated within the electronic platform to support real-time data entry and validation. Refresher training will be provided at least annually and additionally following any material update to registry procedures or variable definitions.

Each user will receive a unique username and password to access the registry through a secure, encrypted connection (TLS 1.2 or higher). User permissions will be role-based, with access restricted according to assigned responsibilities. Participating centers will have access only to their own institutional data and dashboards, while centralized oversight, audit functions, and national-level analytics will remain restricted to authorized SCAR governance personnel.

The platform will generate a unique registry identification code for each patient, while direct patient identifiers will remain stored only at the local participating center in accordance with institutional privacy policies and applicable national data protection regulations. No identifiable patient information will be included in exported analytical datasets. The central database will maintain a complete audit trail documenting data entry, modification, and export activities. Automated daily backups will be stored on encrypted servers to support long-term data preservation and disaster recovery.

Data quality will be supported through standardized variable definitions, structured eCRFs, predefined dropdown menus, automated plausibility and range checks, mandatory field completion prompts, and validation scripts designed to identify missing or inconsistent entries. Regular centralized data reviews and periodic data quality assessments will be conducted to monitor registry performance.

Source-data verification audits will be conducted on a random sample of 5–10% of submitted cases at each participating center per audit cycle to assess data completeness, accuracy, and consistency between registry records and source clinical documentation. A missing data rate exceeding 10% across mandatory fields, or identification of systematic inconsistency during an audit, will trigger a formal corrective action process comprising targeted retraining, data reconciliation, and a follow-up quality review within 60 days. Participating centers will receive periodic feedback reports summarizing data quality indicators, completeness rates, follow-up capture rates, and identified discrepancies to support continuous improvement.

The registry platform will support automated monitoring of missing data, logical inconsistencies, duplicate records, and outlier values. Data quality metrics, including completeness rates, follow-up capture rates, validation-error frequencies, and audit findings, will be reviewed periodically by the registry governance structure to promote adherence to registry standards and ensure reliable outcome reporting across participating institutions.

## 15. Statistical Analysis

Statistical analyses will be performed using R (version 4.4.2; R Foundation for Statistical Computing, Vienna, Austria) or IBM SPSS Statistics (version 29.0; IBM Corp., Armonk, NY, USA). Continuous variables will be summarized as mean ± standard deviation or median with interquartile range, as appropriate, while categorical variables will be presented as counts and percentages.

The analytical framework is organized into four sequential tiers corresponding to the registry’s maturation. First, primary descriptive analyses will characterize patient demographics, comorbidity profiles, operative variables, and unadjusted outcome rates across the registry. These analyses will be performed from the outset of data accrual and will form the basis of annual national reports. Second, risk-adjusted benchmarking analyses will compare institutional outcomes after adjustment for case-mix using predefined clinical variables and established risk factors, with hierarchical models incorporating center-level random effects to account for clustering of patients within participating institutions. Third, validation analyses will evaluate the performance of existing international risk stratification systems, including EuroSCORE II and the STS risk models, within the Saudi cardiac surgical population. These analyses will be undertaken once a nationally representative dataset of sufficient size has been accrued. Fourth, risk prediction model development will be considered only after the minimum event threshold defined in the Risk Prediction Model section has been reached.

Within each tier, specific methods will be applied as follows. Comparisons between groups will be performed using Student’s *t*-test, Mann–Whitney U test, Kruskal–Wallis test, chi-square test, or Fisher’s exact test, as appropriate according to variable type and distribution. Time-to-event outcomes, including survival and freedom from MACCE, will be evaluated using Kaplan–Meier methods and compared using the log-rank test. Multivariable analyses will be conducted using regression models appropriate to the outcome of interest, including logistic regression for binary outcomes, linear regression for continuous outcomes, ordinal regression for ordered outcomes, and Cox proportional hazards models for time-to-event analyses. Propensity score methods, including matching and inverse probability of treatment weighting, will be reserved for specific pre-specified comparative sub-studies rather than applied as a general analytical approach.

Missing data will be evaluated routinely throughout the registry. Where appropriate, multiple imputation by chained equations will be used to address missing data, with complete-case analyses performed as sensitivity analyses to assess the robustness of findings. Patient-reported outcomes will be analyzed using validated scoring methodologies for the EQ-5D and SF-36 instruments. Longitudinal changes in patient-reported outcomes will be evaluated using linear mixed-effects models or generalized estimating equations where sufficient follow-up data are available, with minimally important clinical difference thresholds applied to contextualize observed changes.

Interim descriptive analyses may be performed during early registry implementation to evaluate feasibility, data completeness, follow-up performance, and outcome capture. All statistical tests will be two-sided, and a *p*-value of less than 0.05 will be considered statistically significant.

## 16. Authorship and Data Use

Authorship of publications arising from SCAR will be based on the recommendations of the International Committee of Medical Journal Editors (ICMJE). Authorship eligibility will require substantial contributions to study conception or design, data acquisition, analysis or interpretation, manuscript drafting or critical revision, final approval of the submitted version, and accountability for the integrity of the work.

In recognition of the effort involved in data collection and registry participation, each participating center may nominate up to two investigators for consideration as contributing authors on registry-based publications utilizing their institutional data, provided all ICMJE authorship criteria are fulfilled. The Publication and Dissemination Committee (P&DC) will review and approve authorship lists to ensure fair representation of participating centers and adherence to authorship standards.

Investigators proposing secondary analyses or sub-studies using SCAR data may apply to access registry datasets through a formal proposal process. Proposals will be reviewed by the relevant governance committees and assessed on the basis of scientific merit, methodological rigor, feasibility, and alignment with registry objectives. Investigators leading approved secondary analyses, including protocol development, statistical analysis, and manuscript preparation, may be considered for lead authorship subject to committee approval.

The Steering Committee will oversee preparation of registry-wide reports, approval of analytical datasets, and implementation of data governance policies. Access to de-identified registry datasets for approved research activities will be governed through established data-sharing procedures and applicable ethical approvals. Use of SCAR data for research, audit, or quality improvement activities outside approved registry analyses will require formal proposal submission and approval in accordance with registry governance procedures.

Publication policies, data-access procedures, and authorship principles will be reviewed periodically by the registry governance structure to ensure transparency, fairness, and alignment with evolving registry requirements.

## 17. Dissemination

Findings and recommendations arising from SCAR will be disseminated through regional, national, and international scientific meetings focused on cardiac surgery, cardiovascular medicine, outcomes research, and healthcare quality improvement. Manuscripts generated from registry data will be submitted to peer-reviewed journals relevant to these fields.

In addition to scientific publications, participating centers will receive periodic benchmarking reports and performance summaries through the registry’s secure online dashboard. Annual national reports summarizing patient characteristics, procedural trends, clinical outcomes, benchmarking results, and quality indicators may be produced to support quality improvement initiatives, institutional performance monitoring, and healthcare planning. Aggregated registry findings may also be shared with participating institutions, healthcare authorities, professional societies, and other relevant stakeholders to support quality improvement, healthcare planning, and cardiovascular service development.

Registry findings may be used to inform future quality-improvement initiatives, guideline development, audit activities, and healthcare policy discussions at institutional, regional, and national levels. Periodic dissemination of benchmarking data is intended to facilitate continuous audit-feedback processes and promote evidence-informed improvements in CABG care across participating centers.

All dissemination activities will be conducted in accordance with applicable ethical approvals, data-sharing agreements, and registry governance policies. No individual patients, healthcare professionals, or participating centers will be identifiable in publicly disseminated reports unless explicit permission has been obtained.

## 18. Discussion

Clinical quality registries (CQRs) have emerged as important tools for improving healthcare quality, standardizing clinical practice, facilitating benchmarking, and supporting real-world clinical research. A systematic review demonstrated that most studies evaluating clinical registries reported improvements in healthcare processes and clinical outcomes following registry implementation, with 16 of 17 included studies showing positive effects on quality indicators, adherence to guidelines, or patient outcomes [20]. In cardiovascular medicine and cardiac surgery, registry initiatives have played a central role in outcome surveillance, quality improvement, and evidence generation [21,22,23,24].

Large-scale programs such as the Society of Thoracic Surgeons (STS) Adult Cardiac Surgery Database, the European E-CABG Registry, and the National Adult Cardiac Surgery Audit (NACSA) have demonstrated the value of standardized data collection, risk-adjusted benchmarking, and regular performance feedback in improving surgical quality and supporting clinical research [5,6,7]. These registries have established internationally recognized frameworks for monitoring cardiac surgical outcomes and have contributed substantially to quality assurance initiatives, outcomes research, and evidence-based practice. SCAR builds upon these established principles while adapting them to the clinical, demographic, and healthcare system characteristics of Saudi Arabia.

One of the most important functions of clinical registries is their ability to identify variation in care delivery and outcomes between institutions. National registry systems incorporating audit mechanisms, benchmarking processes, and stakeholder engagement have been associated with measurable improvements in healthcare delivery and reductions in unwarranted practice variation [25]. For a geographically diverse healthcare system such as Saudi Arabia, SCAR may facilitate comparison of outcomes across participating cardiac surgery centers, identify opportunities for quality improvement, and support dissemination of best practices.

Several features may distinguish SCAR from existing international cardiac surgery registries. In addition to capturing conventional perioperative outcomes, the registry incorporates frailty assessment, patient-reported outcome measures, cardiac rehabilitation participation, functional recovery metrics, and structured follow-up at 30 days, 3 months, 6 months, and one year. These elements were intentionally incorporated to provide a broader assessment of recovery following CABG surgery and to align with increasing international emphasis on patient-centered outcomes and value-based healthcare delivery. The dataset further incorporates key echocardiographic parameters, including right ventricular function and aortic valve status, reflecting the established role of multimodality cardiovascular imaging in perioperative risk assessment and long-term outcome prediction [12]. A structured comparison of selected SCAR features with the STS database, E-CABG Registry, and NACSA is presented in Table 2.

The literature consistently highlights several principles required for successful registry implementation, including strong governance structures, standardized data definitions, robust data management systems, user training, quality assurance procedures, and regular feedback to participating centers [27,28,29,30]. These principles are reflected within the SCAR framework through centralized governance, standardized electronic data collection, structured training processes, audit mechanisms, and periodic benchmarking reports. The planned pilot phase will provide an opportunity to evaluate these processes and refine registry operations prior to broader implementation. Such infrastructure is essential for ensuring data quality, maintaining stakeholder engagement, and supporting long-term sustainability.

An additional strength of contemporary registries is their capacity to create a continuous audit-feedback cycle in which collected data are translated into actionable quality-improvement initiatives. Through risk-adjusted benchmarking and longitudinal outcome monitoring, SCAR may support participating centers in identifying performance gaps, evaluating interventions, and monitoring changes in outcomes over time [31].

Modern registry frameworks increasingly emphasize integration with electronic health records, administrative datasets, and advanced analytical platforms [32,33,34]. As the registry matures, opportunities may emerge for linkage with additional healthcare data sources, supporting more comprehensive longitudinal outcome assessment, validation of existing risk prediction models, health services research, and registry-based observational studies. Future development of locally derived risk stratification tools may further enhance the clinical utility of the registry and improve risk estimation within the Saudi population.

Beyond clinical outcome assessment, registries may provide important benefits for healthcare planning and policy development. Registry-derived data can support resource allocation decisions, identify evidence-practice gaps, monitor quality indicators, and inform national cardiovascular health strategies [35,36,37,38]. Within the context of Saudi Arabia’s Vision 2030 healthcare transformation agenda, SCAR may provide a structured framework for monitoring CABG outcomes, supporting quality improvement initiatives, and generating evidence to inform cardiovascular healthcare planning and policy development.

## 19. Limitations

As participation is voluntary, variation in institutional engagement, data completeness, follow-up capture rates, and cardiac rehabilitation reporting may occur across participating centers. Participating institutions may also differ systematically from non-participating centers in case volume, resources, or outcomes, potentially limiting the generalizability of registry findings. Differences in local infrastructure, staffing, and data management capabilities may further contribute to variability in data quality across sites. Although standardized definitions, training programs, automated validation, and audit mechanisms are incorporated into the registry design, the accuracy and completeness of submitted data will ultimately depend on local data collection practices. Variation in local consent requirements may additionally affect case capture rates and registry representativeness.

As an observational registry, SCAR will be subject to the inherent limitations of registry-based research, including residual confounding, missing data, and variation in clinical practice patterns between institutions. Longitudinal follow-up may be affected by loss to follow-up and differential follow-up rates between centers, which could introduce bias into long-term outcome analyses. Development of robust benchmarking systems and locally relevant risk stratification tools will require accrual of sufficiently large datasets with adequate outcome events and sustained multicenter participation. Long-term sustainability will depend on continued institutional commitment, effective governance, robust technical infrastructure, and secure funding support and may be supported through phased implementation, pilot-phase evaluation, and continuous stakeholder engagement.

## 20. Implications for Practice and Research

Once implemented, SCAR may provide a national framework for standardized monitoring of CABG outcomes across Saudi Arabia. Risk-adjusted benchmarking and structured feedback mechanisms may enable participating centers to identify performance variation, support local quality improvement initiatives, and monitor changes in outcomes over time. At a healthcare system level, the registry may generate evidence to support resource allocation, service planning, workforce development, quality assurance activities, and adaptation of clinical practice guidelines to the Saudi healthcare context.

The inclusion of patient-reported outcome measures, functional recovery indicators, and cardiac rehabilitation data may support broader assessment of patient-centered outcomes beyond traditional perioperative metrics. By capturing outcomes across the continuum of care, SCAR may facilitate more comprehensive evaluation of recovery following CABG surgery and promote incorporation of patient-centered measures into routine quality assessment.

In addition, SCAR may serve as a platform for multicenter observational studies, validation of international risk prediction models, development of locally derived risk stratification tools, comparative effectiveness research, and health services investigations. Alignment with internationally recognized data standards may further facilitate collaborative research and strengthen Saudi Arabia’s contribution to global cardiovascular surgery and outcomes research.

By providing a standardized national dataset and longitudinal outcome framework, SCAR may also support future audit initiatives, healthcare quality programs, registry-based research collaborations, and evidence-informed policy development within Saudi Arabia and internationally.

## 21. Key Features of SCAR

While SCAR adopts established design principles from leading international cardiac surgery registries such as the Society of Thoracic Surgeons (STS) Adult Cardiac Surgery Database, the European E-CABG Registry, and the National Adult Cardiac Surgery Audit (NACSA), it incorporates several features intended to address local healthcare priorities and support comprehensive longitudinal outcome assessment within the Saudi healthcare system:

*Comprehensive clinical profiling:* incorporation of frailty assessment, SYNTAX score, healthcare access indicators, cardiac rehabilitation participation, and patient-reported outcome measures alongside conventional perioperative variables.

*Enhanced longitudinal follow-up:* systematic outcome assessment at 30 days, 3 months, 6 months, and one year to facilitate evaluation of recovery trajectories beyond traditional perioperative endpoints.

*Patient-reported outcome measures:* integration of baseline and longitudinal EQ-5D and SF-36 assessments to evaluate health-related quality of life, functional recovery, and patient-centered outcomes following CABG surgery.

*Cardiac rehabilitation and recovery monitoring:* inclusion of referral, participation, and completion of cardiac rehabilitation programs together with return-to-work status and recovery of baseline functional activity.

*Risk-model validation and development:* prospective collection of variables required for evaluation of established international risk prediction models and future development of locally derived risk stratification tools.

*Real-time feedback and benchmarking tools:* secure institutional dashboards providing outcome reporting, data completeness metrics, follow-up performance indicators, and risk-adjusted benchmarking to support continuous quality improvement.

*Nationally standardized data framework:* harmonized definitions, structured data collection processes, and centralized governance to facilitate consistency across participating centers and support future interoperability with national healthcare information systems.

These features position SCAR not only as a national quality-improvement initiative but also as a platform for cardiovascular outcomes research, healthcare planning, risk-model validation, and international collaboration.

## 22. Future Expansion

Following the establishment of standardized data collection processes and broader institutional participation, the registry may be expanded to include additional cardiac surgical procedures beyond CABG, including valve surgery, aortic surgery, and combined interventions. The long-term vision is to integrate SCAR within the national health information infrastructure, where it can serve as a continuous platform for quality improvement, national benchmarking, outcomes research, and multicenter collaborative studies across adult cardiac surgery in Saudi Arabia. In addition to supporting clinical audit and quality improvement, future iterations of the registry may facilitate linkage with national health databases, mortality records, and healthcare utilization datasets to enable more comprehensive longitudinal outcome assessment and health services research. Opportunities may also emerge for integration with other national cardiovascular datasets, supporting broader evaluation of patient pathways and long-term outcomes across the continuum of cardiac care. As registry participation expands and data maturity increases, SCAR may also support validation of emerging risk prediction models, registry-based observational studies, comparative effectiveness research, and future quality-improvement initiatives across the Saudi cardiovascular care system.

## 23. Conclusions

The Saudi CABG Audit and Registry (SCAR) represents a national initiative to support standardized monitoring of perioperative and long-term outcomes following coronary artery bypass grafting in Saudi Arabia. Drawing upon established principles from leading international cardiac surgery registries, SCAR provides a structured framework for capturing clinical, operative, rehabilitation, and patient-reported outcomes across the continuum of care, from preoperative assessment to long-term recovery. Through standardized data collection, risk-adjusted benchmarking, and longitudinal outcome assessment, SCAR may support quality improvement initiatives, evidence-informed clinical decision-making, healthcare planning, and multicenter cardiovascular research. The registry also provides a platform for evaluation of existing risk stratification systems, validation of international risk prediction models, future development of locally derived risk stratification tools, and generation of real-world evidence to inform cardiac surgical practice. Successful implementation, sustained stakeholder engagement, high-quality data capture, and continued institutional participation will be essential to realizing the full potential of the registry and supporting ongoing improvements in cardiac surgical care within Saudi Arabia.

## Figures and Tables

**Table 1:** SCAR data collection parameters and time points.

Time Point	Domain	Key Variables Collected
Admission/Preoperative	Demographics and Risk Factors	National ID (anonymized), age, sex, height, weight, BMI, nationality, smoking status (current/former/never), hypertension, dyslipidemia, diabetes mellitus (type, treatment modality, HbA1c), chronic kidney disease (KDIGO stage) [11], dialysis, renal transplant, chronic lung disease, extracardiac arteriopathy, prior PCI (date, vessel, stent type, complications), prior CABG, prior myocardial infarction, frailty scale (CSHA) [13], poor mobility, employment status (employed/unemployed/retired)
	Cardiac Status and Risk Scores	LVEF, NYHA class [14], CCS angina class [15], recent MI (≤30 days), Killip class [16], baseline functional capacity, ventricular arrhythmia history, atrial fibrillation, SYNTAX score [10], GRACE score [17], EuroSCORE II [9], left main stenosis, number of diseased vessels, preoperative heart rate, preoperative blood pressure, mitral regurgitation grade, tricuspid regurgitation grade, pulmonary artery systolic pressure, right ventricular function (TAPSE), aortic valve disease severity, baseline EQ-5D, baseline SF-36
	Preoperative Medication Profile	Aspirin, P2Y12 inhibitor, oral anticoagulant therapy, statin therapy, beta-blocker, ACE inhibitor/ARB, SGLT2 inhibitor, GLP-1 receptor agonist, preoperative antibiotic therapy (non-prophylactic), date of antithrombotic discontinuation
Intraoperative	Operative Data	Date/time of surgery, surgical urgency (elective, urgent, emergency class 1–4), revascularization technique (on-pump, off-pump, HB-CPB, conversions), CPB use, CPB time, aortic cross-clamp time, graft type (arterial, venous, composite), number of grafts performed, number of distal anastomoses, cardioplegia type and temperature, epiaortic ultrasound, ascending aorta status, porcelain aorta, off-pump surgeon experience, concomitant Maze procedure, intraoperative blood product transfusion, intraoperative complications
Postoperative (In-Hospital)	In-Hospital Outcomes	ICU length of stay, prolonged ICU stay, hospital length of stay, discharge destination (home, rehabilitation facility, long-term care facility, other), duration of mechanical ventilation, re-intubation, myocardial infarction (Type V definition) [18], stroke (permanent/temporary, imaging-confirmed), deep sternal wound infection (CDC criteria) [19], leg wound infection, mediastinitis, reoperation (bleeding, tamponade, graft failure), early repeat revascularization (PCI/CABG), new-onset atrial fibrillation, ventricular arrhythmia, pericardial effusion requiring drainage, AKI (KDIGO), new renal replacement therapy, delirium requiring medication, VTE (deep vein thrombosis/pulmonary embolism), nadir Hb/Hct, blood loss in first 12 h, blood products transfused, prolonged inotropes (>12 h), postoperative IABP, ECMO, in-hospital mortality
30 Days	Follow-up Outcomes	All-cause mortality, wound complications, readmission (all-cause), MACCE, medication adherence, NYHA class, cardiac rehabilitation referral, cardiac rehabilitation participation
3 Months	Follow-up Outcomes	Recovery status, persistent symptoms (angina, dyspnea), medication adherence, wound healing, NYHA class, cardiac rehabilitation participation, return-to-work status (where applicable), EQ-5D, SF-36
6 Months	Follow-up Outcomes	Functional recovery (NYHA class), return to routine activity, interim MACCE, physical activity level, medication adherence, cardiac rehabilitation completion status, return-to-work status (where applicable), EQ-5D, SF-36
1 Year	Follow-up Outcomes	Survival (all-cause and cardiovascular), MACCE, repeat revascularization, late myocardial infarction, late stroke, cardiovascular mortality, medication adherence, NYHA class, return-to-work status (where applicable), cardiac rehabilitation completion status, EQ-5D, SF-36, change in EQ-5D from baseline, change in SF-36 from baseline, minimal clinically important difference (MCID) analysis

**Table 2:** Comparison of selected features of the Saudi CABG Audit and Registry (SCAR) with major international cardiac surgery registries [5,6,7,26]. Registry characteristics are based on publicly available registry descriptions and published literature and may not reflect all features or recent updates. Feature availability may vary between participating institutions and over time. STS ACSD, Society of Thoracic Surgeons Adult Cardiac Surgery Database; E-CABG, European Multicenter Study on Coronary Artery Bypass Grafting; NACSA, National Adult Cardiac Surgery Audit; CSHA, Canadian Study of Health and Aging Clinical Frailty Scale; TAPSE, tricuspid annular plane systolic excursion; NHS, National Health Service. (✓)—Present.

Feature	STS ACSD	E-CABG Registry	NACSA	SCAR
Geographic scope	National (USA)	Multinational (Europe)	National (UK)	National (Saudi Arabia)
Procedures covered	All adult cardiac surgery	Isolated CABG	All adult cardiac surgery	CABG (isolated and combined)
Participation	Voluntary	Voluntary	Mandatory (NHS)	Voluntary
Validated risk model	✓ (STS-PROM)	✓ (EuroSCORE II)	✓ (EuroSCORE II)	Planned
Frailty assessment	Partial	Not routinely	Partial	✓ (CSHA)
Structured longitudinal follow-up	30-day/Medicare linkage	Up to 10 years	30-day in-hospital	✓ (30 days, 3, 6, 12 months)
Patient-reported outcomes	In development	Not routinely	Not routinely	✓ (EQ-5D, SF-36)
Cardiac rehabilitation capture	Not routinely	Not routinely	Not routinely	✓
Return-to-work status	Not routinely	Not routinely	Not routinely	✓
Echocardiographic parameters	Selected	Selected	Selected	✓ (incl. TAPSE, aortic valve)
Real-time institutional dashboards	✓	Variable	✓	✓
Source-data verification audit	✓	Variable	✓	✓ (5–10% random)

## Data Availability

No data were collected or generated in association with this manuscript, which describes a registry protocol prior to implementation.
